# The immunomodulator PSK induces *in vitro *cytotoxic activity in tumour cell lines *via *arrest of cell cycle and induction of apoptosis

**DOI:** 10.1186/1471-2407-8-78

**Published:** 2008-03-24

**Authors:** Eva Jiménez-Medina, Enrique Berruguilla, Irene Romero, Ignacio Algarra, Antonia Collado, Federico Garrido, Angel Garcia-Lora

**Affiliations:** 1 Servicio de Análisis Clínicos e Inmunologia, Hospital Universitario Virgen de las Nieves, Universidad de Granada, Av. de las Fuerzas Armadas 2, 18014 Granada, Spain; 2 Departamento de Ciencias de la Salud, Universidad Jaén, Jaén, Spain; 3 Unidad de Investigación, Hospital Universitario Virgen de las Nieves, Granada, Spain

## Abstract

**Background:**

Protein-bound polysaccharide (PSK) is derived from the CM-101 strain of the fungus *Coriolus versicolor *and has shown anticancer activity *in vitro *and in *in vivo *experimental models and human cancers. Several randomized clinical trials have demonstrated that PSK has great potential in adjuvant cancer therapy, with positive results in the adjuvant treatment of gastric, esophageal, colorectal, breast and lung cancers. These studies have suggested the efficacy of PSK as an immunomodulator of biological responses. The precise molecular mechanisms responsible for its biological activity have yet to be fully elucidated.

**Methods:**

The *in vitro *cytotoxic anti-tumour activity of PSK has been evaluated in various tumour cell lines derived from leukaemias, melanomas, fibrosarcomas and cervix, lung, pancreas and gastric cancers. Tumour cell proliferation *in vitro *was measured by BrdU incorporation and viable cell count. Effect of PSK on human peripheral blood lymphocyte (PBL) proliferation *in vitro *was also analyzed. Studies of cell cycle and apoptosis were performed in PSK-treated cells.

**Results:**

PSK showed *in vitro *inhibition of tumour cell proliferation as measured by BrdU incorporation and viable cell count. The inhibition ranged from 22 to 84%. Inhibition mechanisms were identified as cell cycle arrest, with cell accumulation in G_0_/G_1 _phase and increase in apoptosis and caspase-3 expression. These results indicate that PSK has a direct cytotoxic activity *in vitro*, inhibiting tumour cell proliferation. In contrast, PSK shows a synergistic effect with IL-2 that increases PBL proliferation.

**Conclusion:**

These results indicate that PSK has cytotoxic activity *in vitro *on tumour cell lines. This new cytotoxic activity of PSK on tumour cells is independent of its previously described immunomodulatory activity on NK cells.

## Background

A number of bioactive molecules, including antitumour substances, have been identified in various mushroom species. Polysaccharides are the best known and most potent of these and have antitumour and immunomodulating properties [1-5]. PSK, a protein-bound polysaccharide obtained from *Basidiomycetes*, also known as *Krestin*, has been used as an agent in the treatment of cancer in Asia for over 30 yrs [6-8]. PSK is derived from the fungus *Coriolus versicolor *and has documented anticancer activity *in vitro *in experimental models [9] and in human clinical trials. Several randomized clinical trials have demonstrated that PSK has great potential in adjuvant cancer therapy, with positive results in the treatment of gastric, esophageal, colorectal, breast and lung cancers [10,11]. These studies have suggested the efficacy of PSK as an immunomodulator of biological response.

Previous reports indicated that PSK might act in different ways: as antioxidant [5,12,13]; as inhibitor of metalloproteinases and other enzymes involved in metastatic processes [14] and as inhibitor of the action of various carcinogens in vulnerable cell lines. However its most important and widely reported property is its immunomodulatory capacity. PSK may act to increase leukocyte activation and response *via *upregulation of key cytokines. Thus, natural killer (NK) and lymphocyte-activated killer (LAK) cell activation has been demonstrated *in vivo *and *in vitro *[15,16]. Our group demonstrated that PSK is capable of inhibiting metastatic colonization *in vivo *in some experimental fibrosarcomas, and that this effect is mediated by activation of NK cells [17,18]. Moreover, the NK cell line NKL, derived from a large granular lymphocyte leukaemia [19], is activated *in vitro *by PSK [16]. This activation may replace IL-2 in inducing the proliferation and cytotoxicity of NKL cells. The signal transduction pathways involved in the responses to IL-2 or PSK are different: IL-2 increases PKCα and ERK3 expression and decreases ERK2 expression, whereas PSK decreases PKCα expression and increases ERK3 expression [20]. PSK also enhances CRE binding activity, while IL-2 increases SP-1 and modifies GAS/ISRE, IRF-1 and STAT5 [21]. In addition, PSK and IL-2 have been shown to bind to different receptor on NKL cells [22].

The direct *in vitro *effect of PSK on the proliferation of tumour cell lines was compared with its effect on PBLs. PSK had cytotoxic activity on tumour cell lines, inhibiting proliferation, producing cell cycle arrest and cell accumulation in G_0_/G_1_phase and inducing apoptosis.

## Methods

### Protein-bound polysaccharide K

Protein-bound polysaccharide K (PSK) was kindly provided by Kureha Chemical Ind. Co. (Tokyo, Japan). It is prepared by extracting cultured mycelia of *Coriolus versicolor *with hot water. The precipitate is separated from the clear supernatant with saturated ammonium sulfate, then desalted and dried [23]. Protein-bound polysaccharide K was dissolved in RPMI medium or water and heated at 50°C for 20–30 min until a clear solution appeared. The PSK preparation was filter-sterilized and diluted in culture medium or water to the desired concentration. Protein-bound polysaccharide K was previously titrated in NKL cells [16] and the working dilution was 100 μg/mL. PSK extract digested with neuraminidase was also tested, digesting 100 μg of PSK with 4 μl (Sigma) and incubating for 3 h at 37°C. Our group previously showed that PSK is composed of two bands of very high molecular weight [22]. After digestion with neuraminidase, these bands are reduced to a single band of about 12 kd. These results indicate that PSK is probably composed of a single 12-kd protein, and that this protein is highly glycosylated [22]. Two different extracts of PSK were also used: one rich in sugars and other rich in proteins.

### Cell lines and cell culture

The following tumour cell lines were studied: B16 murine melanoma, B9 murine MCA-induced fibrosarcoma, Ando-2 human melanoma, AGS human gastric cancer, A-549 human lung cancer, Hela human cervical adenocarcinoma and Jurkat T lymphoma leukemia. The NKL studied was established from PBLs of a patient with LGL leukemia [19]. All cell lines were obtained from the American Type Culture Collection (Manassas, USA) except for the B9 cell line, which was generated at our laboratory, and the Ando-2 and NKL cell lines, kindly provided by P. Coulie (Unite de Genetique Cellulaire, Louvain University, Brussels, Belgium), F. X. Real (Instituto Municipal de Investigaciones Medicas, Barcelona, Spain) and Dr. M. Lopet-Botet (Universidad Pompeu-Fabra, Barcelona, Spain), respectively.

Cell lines derived from solid tumours were grown at 37°C in a humidified atmosphere of 5% CO_2 _in DMEM culture medium (Gibco, Paisley UK) supplemented with 10% heat-inactivated foetal bovine serum (Life Technologics, Milan Italy), antibiotics and glutamine. Jurkat T cell leukemia was cultured in RPMI 1640 with 10% heat-inactivated fetal bovine serum. The NKL cell line was cultured in RPMI 1640 with 10% heat-inactivated human AB serum (Sigma Chemical, St Louis, MO; USA) and human recombinant IL-2 (100 U/ml; purity > 97%, specific activity, 2 × 10^6 ^U/mg) (Roche, Nutley, NJ; USA).

### In vitro cytotoxicity assays

The effect of PSK on tumour cell proliferation was assessed by measuring BrdU incorporation with the BrdU colorimetric ELISA Cell Proliferation Kit (Roche Diagnostic). Cells were plated in 96-well microculture plates (5 × 10^3 ^cells/well). Every 48 h, the culture medium was replaced and PSK was added. After 48–96 h, BrdU labelling reagent was added and cultured for a further 1–3 h. Assays were also performed by counting viable cells using Trypan Blue. Briefly, cancer cell lines were seeded into culture tissue-flask (1.5–2 × 10^5^/culture tissue-flask) and incubated for 24 h at 37°C in a humidified atmosphere of 5% CO_2_. Cells were then treated with 100 μg/ml of PSK in the culture medium, which was replaced every 48 h. After 4–6 days, cells were collected by centrifugation and a small sample of cell suspension was diluted in 0.4% Trypan Blue, counting cells in a haemocytometer chamber. Each cell sample was counted in this way at least three times and each assay was repeated at least three times.

### Lymphocyte and NKL proliferation assay

Human lymphocytes were isolated from venous blood by the Ficoll-Hystopaque separation method. Proliferation of PBLs was analyzed *in vitro *using 5-bromo-2'-deoxyuridine (BrdU) labelling of DNA-synthesizing cells with the above-mentioned kit. PBLs were seeded in 96-well microculture plates at a cell density of 5 × 10^4 ^per well. Two different concentrations of PSK were used, 100 μg/ml and 50 μg/ml. Concanavalin A (5 μg/ml, Sigma) and IL-2 were used as positive controls. PSK was also used in combination with IL-2 or Concanavalin A. After 48 h of culture in presence or absence of PSK, BrdU labelling reagent (final concentration 10 μM) was added and cells were cultured for 24 h. Cells were then fixed for 30 min and incubated with anti-BrdU for 1 h at 37°C. 100 μl of tetramethyl-benzidine (TMB) was used as substrate. Optical densities were determined at 370 nm by means of an ELISA microplate reader (Biotek, Power-Wave XS). Controls were the culture medium, cells cultured only in medium and cells incubated with anti-BrdU in absence of BrdU. All experiments were repeated at least three times.

### Cell cycle distribution analysis

Briefly, cells were plated in six-well plates (5 × 10^5 ^per well) or in culture tissue-flask (15 × 10^5^) and continuously exposed for 4 days to 100 μg/ml of PSK. The DNA synthesis rate was examined by BrdU incorporation method using FITC BrdU Flow Kit (BD Pharmingen) according to manufacturer's instructions. BrdU was then detected by DNase cell treatment using FITC-conjugated anti-BrdU antibody. Cells were washed with 1 ml 1 × BD Perm/Wash Buffer, and 20 μl 7-amino-actinomycin D was added. Analysis was performed with 50000 cells using Cell Quest Software and FACScan flow cytometer (Becton-Dickinson).

### Annexin V binding assay to detect apoptotic cells

After treatment of cancer cells with PSK for four days, cells were detached from the culture tissue-flask with PBS containing 3 mM EDTA. These cells were then collected together with floating cells, washed twice with cold PBS and resuspended in binding buffer at a concentration of 1 × 10^6 ^cells per ml; 100 μl of solution was incubated for 30 min at 4°C with 5 μl of Annexin V-PE antibody (BD Biosciences), and 5 μl of 7-amino-actynomycin D was then added. Cells were incubated for 15 min in darkness, and 400 μl of staining buffer was added before flow cytometry analysis. Apoptosis was analyzed by quadrant statistics as follows: Annexin V- and 7-AAD-negative cells are alive; Annexin V-positive and 7-AAD-negative cells are in early stages of apoptosis; Annexin V-negative and 7-AAD-positice cells are dead but not by apoptosis; and Annexin V-positive and 7-AAD-positive cells are in mid- or end-stage apoptosis.

### Assay for active caspase-3 expression

FITC conjugated monoclonal anti-active-caspase-3 antibody (BD Biosciences) was used to determine whether the protease caspase-3 is involved in PSK-induced apoptosis. After 4-day treatment with PSK, cancer cells were washed twice with cold PBS and fixed and permeabilized in Cytofix/Cytoperm buffer. Then, cells were incubated with FITC-conjugated monoclonal rabbit anti-active human-caspase-3 antibody for 30 min. Cells were washed twice and 500 μl of 1 × Perm Wash Buffer was added before analysis by flow cytometry.

### Statistical analysis

Values are expressed as means ± SD. Student's *t*-test was used for statistical comparisons, considering a significance value of P < 0.05.

## Results

### PSK inhibits *in vitro *tumour cell proliferation

**Figure 1:**
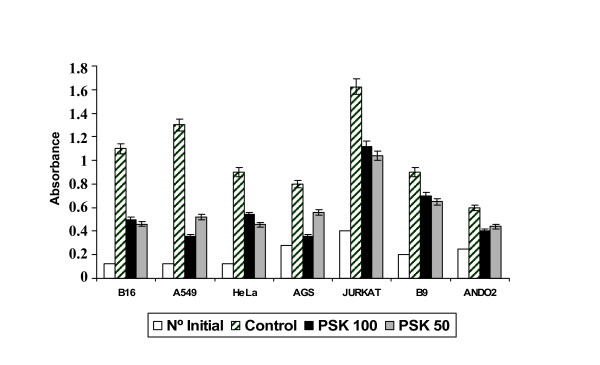
**Effect of PSK on tumour cell line proliferation**. B16, A549, Hela, AGS, Jurkat, B9 and Ando-2 tumour cell lines were treated or not with 50 μg/ml or 100 μg/ml of PSK for 72–96 h. Tumour cells (2.5 to 5 × 10^4^) were plated in quadruplicate in 96-well plates. The proliferation of tumour cells was determined by BrdU incorporation and absorbance measurement. All cell lines analysed showed inhibition of proliferation. Each column represents the mean of five independent experiments ± SD. P < 0.001 versus control.

Tumour cell lines were cultured in 96-well plate for 48–72 h (2.5–5 × 10^3 ^cells) in medium alone (control) or with PSK (100 μg/ml or 50 μg/ml) for 4–6 days. Cell proliferation was then measured by BrdU incorporation (absorbance), which was significantly lower in PSK-treated versus untreated tumour cells (Fig. 1). AGS and A549 cell lines showed a strong decrease in absorbance after treatment with 100 μg/ml PSK that was less marked after treatment with 50 μg/ml of PSK. Inhibition of proliferation was around 65% in melanoma cell lines B16 and Ando-2, lower in Hela and Jurkat cell lines and lowest (20%) in B9 murine fibrosarcoma (Fig. 1). PSK-treated tumour cells showed morphological changes (rounded and granulated morphology, increased vacuolisation, cell shrinkage) and a large number of the cells detached from culture flasks.

These assays were repeated in cell culture flasks (1.5–2.5 × 10^5^cells), and viable cells were counted in Haemacytometer chamber using Trypan Blue. As shown in Table 1, there was a significant decrease in the final number of viable cells, with a proliferation inhibition of 22%–84% versus control cells. There was an excellent correlation between the results obtained with the two assays (absorbance and cell count). In Hela tumour cells, proliferation inhibition was higher after treatment with 50 μg/ml versus 100 μg/ml of PSK. In all other tumour cell lines, proliferation inhibition was similar or higher at 100 μg/ml PSK.

**Table 1:** Proliferation of tumour cell lines treated with PSK

	**Cell No. (× 10^4^)**				**% inhibition**	
		
**Tumour cell line**	**Initial**	**Control**	PSK1*	PSK2*	**PSK1**	**PSK2**
B16	25	220 ± 5	100 ± 2	92 ± 2	62	65
AGS	14	40 ± 1	18 ± 1	21 ± 1	84	71
Hela	25	180 ± 5	108 ± 4	91 ± 4	46	57
A549	25	260 ± 5	72 ± 2	104 ± 3	80	56
Jurkat	80	325 ± 5	224 ± 3	208 ± 3	41	47
B9	20	90 ± 4	75 ± 3	72 ± 3	22	26
Ando-2	15	30 ± 1	20 ± 1	22 ± 1	67	54

*PSK1: 100 μg/ml; PSK2: 50 μg/ml. P < 0.001 versus control

### PSK increases *in vitro *proliferation of IL-2-stimulated lymphocytes

**Figure 2:**
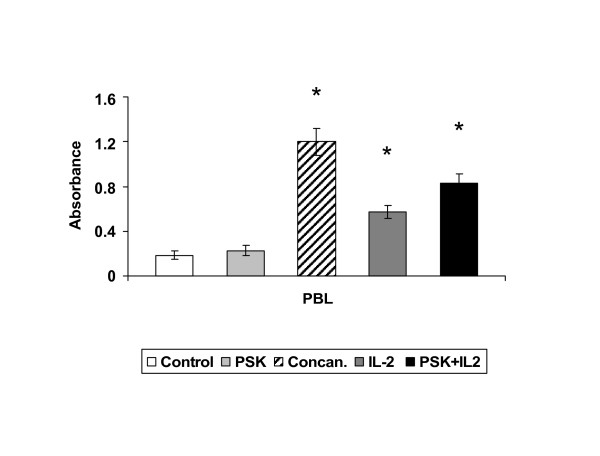
**Effect of PSK on PBL proliferation**. PBLs were cultured with PSK or IL-2 or with PSK+IL-2. PBLs (50 × 10^4 ^cells) were seeded in quadruplicate in 96-well plates. The proliferation was determined by BrdU incorporation and absorbance measurement. PSK showed a synergistic effect with IL-2, increasing PBL proliferation. PSK alone did not induce PBLs proliferation. Each column represents the mean of five independent experiments ± SD. *P < 0.001 versus control.

A dose-response analysis was performed to determine the *in vitro *effect of PSK on human PBLs. PBLs (5 × 104) were plated in 96-well tissue plate for 48–72 h with eight serially diluted extractions ranging from 500 μg/ml (concentration n°8) to 3.9 μg/ml (concentration n°1). Concentration n°0 represents cells cultured in medium alone. BrdU incorporation during DNA synthesis was then measured by ELISA. Optical densities were very similar between treated and untreated PBLs (data not shown). However, simultaneous treatment of PBLs with IL-2 (100 U/ml) + PSK (100 μg/ml) produced a higher proliferation rate (4.5-fold) versus PBLs treated with IL-2 alone (3-fold) (Fig. 2). Untreated and Concanavalin A-treated PBLs served as controls (Fig. 2).

### Effect of different variants of PSK

**Figure 3:**
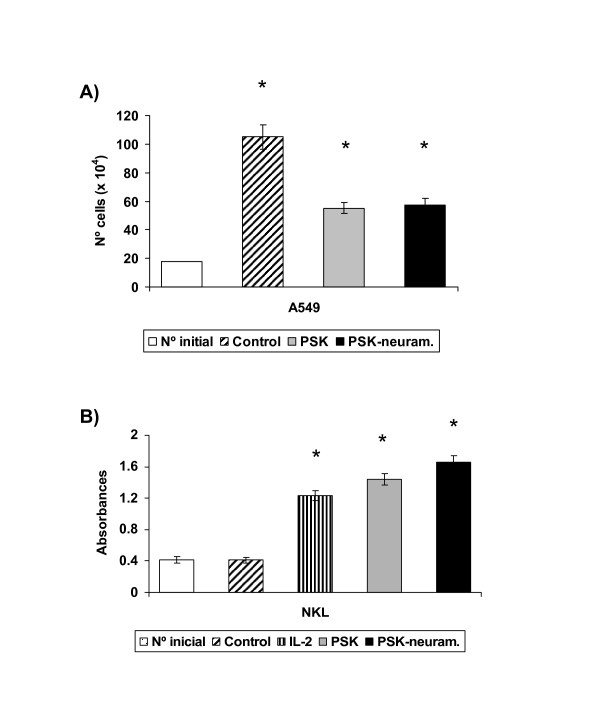
**In vitro activity of neuraminidase treated-PSK**. **a) **A549 tumour cell line was cultured with neuraminidase-treated PSK or PSK. A549 tumour cells (20 × 10^4^) were seeded in culture flask and treated with PSK for 96 h, estimating cell viability by means of Trypan blue exclusion. Both agents produced a similar inhibition ofproliferation. **b) **NKL cell line was cultured with PSK or neuraminidase treated-PSK and proliferation was determined by BrdU incorporation and absorbance measurement. Both agents induced a similar increase of proliferation. Each column represents the mean of five independent experiments ± SD. *P < 0.001 versus control.

Tumour cell proliferation inhibition was compared among different PSK variants. Neuraminidase treatment digests glicosylated proteins. A549 tumour cell line was cultured in medium alone (control) or with PSK (100 μg/ml) or neuraminidase-treated PSK (100 μg/ml) for 4–6 days and then counted using trypan blue. No significant differences in proliferation inhibition were found between PSK and neuraminidase-treated PSK (Fig. 3a). The same results were found for sugar-rich and protein-rich PSK variants as for PSK (data not shown).

It was previously reported that PSK induces proliferation and activation of NKL cells [16]. Treatment of NKL cells with 100 μg/ml PSK or neuraminidase-treated PSK for 96 h induced a similar increase in their proliferation (Fig. 3b), which was slightly higher than that obtained after culture of NKL with IL-2 alone (Fig. 3b). Induction of NKL proliferation was slightly lower in sugar-rich and protein-rich PSK variants; this difference was not significant (data not shown)

### Cell cycle phase distribution analysis of PSK-treated cells

**Figure 4:**
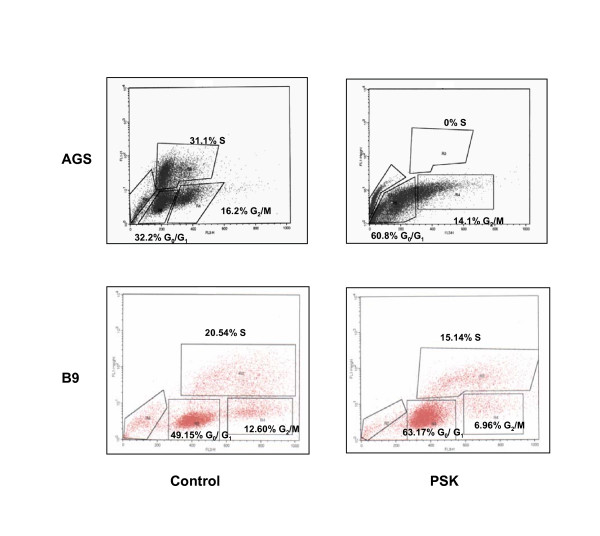
**Cell cycle analysis of cells treated with PSK**. Tumour cell lines were treated with PSK for 96 h. Cell cycle distribution was determined by flow cytometry using BrdU incorporation and 7-AAD. Data indicate the percentage of cells in each phase of cell cycle. Results are representative of three independent experiments.

Mechanisms of PSK cytotoxic activity were analysed by flow cytometry in order to study the effect on cell cycle phase distribution. Culture of AGS tumour cell line with 100 μg/ml of PSK produced total cell cycle arrest with cell accumulation in G_0_/G_1 _phase and no cells in S phase (Fig. 4). Cell cycle phase distributions were: 32.2% G_0_/G_1_, 31.1% S and 16.2% G_2_/M in control AGS cells and 60.8% G_0_/G_1_, 0% S and 14.1% G_2_/M in PSK-treated AGS cells. Similar results were found in Ando-2, A549 and B16 tumour cell lines (Table 2). Results in B9 fibrosarcoma showed a slowing rather than an arrest of the cell cycle (Fig.4), with a partial accumulation in G_0_/G_1 _phase (49.15% untreated cells and 63.17% PSK-treated cells) at the expense of a decrease in S phase (20.54% vs. 15.14%) and G_2_/M phase (12.6% vs. 6.96%). Similar results were found in Hela and Jurkat tumour cells (Table 2). These results indicate that PSK produces arrest or slowing of the cell cycle according to the tumour cell histology.

**Table 2:** Effect of PSK on cell cycle distribution of tumor cell lines.

**Tumour cell line**	**Cell cycle distribution (%)**			
	
	**SUB-G1**	**G2**	**S**	**G1**
AGS control	5.12	16.20	31.10	32.20
AGS PSK	12.23	14.10	0	60.80
Ando-2	6.35	18.12	32.25	33.46
Ando-2 PSK	12.40	16.30	4.35	60.10
A549 control	7.25	15.53	31.15	30.67
A549 PSK	11.95	13.13	5.20	49.45
B16 control	8.35	12.25	30.23	31.45
B16 PSK	14.35	8.35	5.45	55.67
Jurkat control	8.23	18.60	32.35	25.50
Jurkat PSK	11.30	18.35	15.25	45.35
HELA control	5.58	16.35	21.12	44.67
HELA PSK	10.57	9.56	14.78	51.56
B9	5.20	12.60	20.54	49.15
B9 PSK	6.56	6.96	15.14	63.17

Results are for one typical experiment out of three performed.

### Analysis of apoptosis in tumour cells treated with PSK

**Figure 5:**
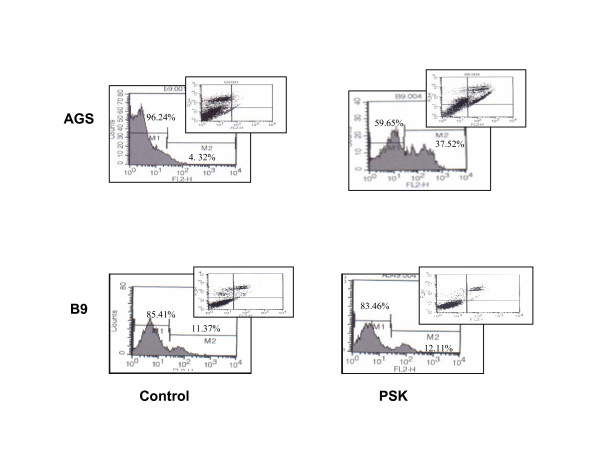
**Apoptosis analysis of cells treated with PSK**. AGS cell line was untreated or treated with 50 μg/ml of PSK for 96 h. Cells were double-stained with annexin V and 7AAD and analyzed by flow cytometry. PSK produced apoptosis in AGS tumour cell line. B9 tumour cell line was also cultured with PSK but apoptosis was not detected in this tumour cell line. All experiments were performed at least three times and gave similar results.

Cancer cell lines were treated with 100 μg/ml PSK for 4 days to examine the capacity of PSK to induce apoptosis. Untreated or PSK-treated cancer cells were incubated with Annexin V-PE in a buffer containing 7-amino-actinomycin (7-AAD) and analyzed by flow cytometry. Figure 5 depicts representative results for AGS and B9 tumour cells. PSK increased apoptosis from 4.32% (untreated cells) to 37.52% in AGS cells but not in B9 tumour cells (untreated cells, 11.37% vs. PSK-treated cells, 12.11%). Table 3 depicts the results for other tumour cell lines, showing that PSK induces apoptosis in A549, B16 and Ando-2 tumour cells.

**Table 3:** Apoptosis induction in cancer cell lines after treatment with PSK for 4 days.

**Tumour cell line**	**Apoptosis (%)**	
	
	**Control**	**PSK 100 μg/ml**
AGS	4.32%	37.52%
A549	10.40%	35.50%
B16	8.90%	25.40%
Ando-2	7.98%	18.35%
JURKAT	6.10%	10.71%
HELA	9.35%	14.30%
B9	11.37%	12.11%

This assay was repeated at least three times, producing similar results.

### Expression of active human caspase-3

**Figure 6:**
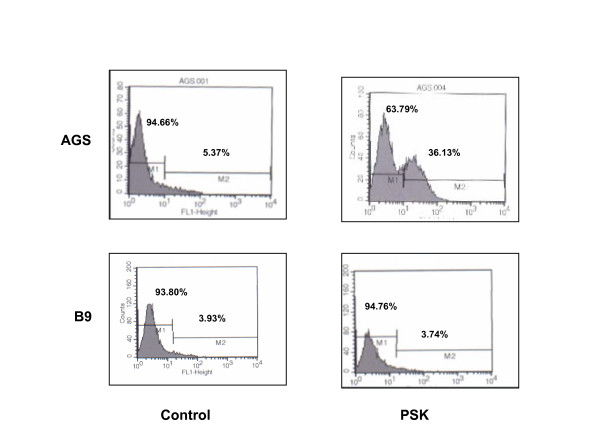
**Caspase-3 expression in tumour cell lines treatedwith PSK**. AGS and B9 tumour cell lines were treated with 50 μg/ml of PSK and expression was analysed by flow cytometry using FITC conjugated monoclonal anti-active-caspase-3 antibody. Data indicate the percentage of cells positive for presence of active-caspase-3. PSK produced increased caspase-3 expression in AGS but not in B9 tumour cell lines. Results are representative of three experiments.

Caspases are the main enzymes involved in the apoptotic pathway and the participation of active caspase-3 in PSK-induced apoptosis was evaluated. Tumour cells were treated with PSK (100 μg/ml) for 4 days, then permeabilized, fixed and stained for active human caspase-3 and analyzed by flow cytometry. In the AGS cell line, untreated cells were negative for presence of active-caspase-3, whereas around 36% of PSK-treated cells showed detectable active caspase-3 (Fig. 6). However, in tumour cell lines in which PSK did not produce apoptosis, e.g., B9 tumour cells, no caspase-3 expression was detected after PSK treatment (Fig. 6). Table 4 depicts the results obtained with the other tumour cell lines analysed.

**Table 4:** Expression of caspase-3 in cancer cell lines after treatment with PSK for 4 days.

**Tumour cell line**	**Expression (%)**	
	
	**Control**	**PSK100 μg/ml**
AGS	5.37%	36.13%
A549	4.20%	31.14%
B16	6.80%	23.35%
Ando-2	5.40%	15.55%
JURKAT	5.35%	6.45%
HELA	6.50%	6.15%
B9	3.93%	3.74%

This assay was repeated at least three times, producing similar results

## Discussion

Several clinical assays have reported the anti-tumour properties of PSK and its synergestic effect in combined therapies [9,24,25]. Our group previously reported the immunomodulatory activity of PSK on NK cells, producing *in vitro *proliferation and activation of NKL cells [16,20,21]. In the present study, we have identified a new cytotoxic anti-tumour activity of PSK. This activity varied according to the histological origin of the tumour cell lines under study, with inhibition rates ranging from 84% to 22% (Table 1). The highest profileration inhibition rates were found in AGS (84%) and A549 (80%) cell lines (gastric and lung cancer, respectively). PSK was previously reported to be effective in adjuvant immunotherapy for patients after curative resection of gastric cancer [25], and this effect was attributed to its immunomodulatory activity on NK cells [26]. Our group previously reported that PSK mediates induction of the NKL cell proliferation and activation. The present results suggest that PSK may also exert a direct antitumour cytotoxic activity. Inhibition was around 65% in melanoma cell lines Ando-2 (human) and B16 (mice) and was lowest (22%) in the B9 murine fibrosarcoma cell line. Deglycosylation of PSK by neuraminidase treatment did not modify its cytotoxic effect on tumour cell lines. The sugar-rich and protein-rich PSK variants showed identical results to those of PSK in their inhibition of proliferation of tumour cell lines *in vitro*. These results indicate that the cytotoxic properties are in a compound that is present in all three variants studied and does not vary among them.

Interestingly, PSK had the opposite effect on lymphocytes. Thus, PSK, in synergy with IL-2, induced proliferation of PBLs. PSK also induced proliferation and activation of NKL cells, producing an effect similar to that of IL-2. Hence, PSK has a cytotoxic effect on tumour cells and a mitotic effect on lymphocytes and NK cells.

The cell cycle was arrested or slowed by PSK according to the histological origin of the tumour cells. PSK is known to increase docetaxel-induced apoptosis of NOR-P human pancreatic cancer cells [27] and of Namalwa Burkitt lymphoma cells [28]. PSK induced apoptosis in the AGS cell line but not in all tumour cell lines analysed and induced caspase-3 expression in some tumour cell lines but not all. These results indicate that PSK may induce cytotoxic activity by different molecular mechanisms according to the histology of tumour.

The molecular mechanisms implicated in PSK-induced proliferation and activation of NKL cells have been widely described, showing that PSK and IL-2 bind to different receptors on NKL cells and induce different signal transduction pathways [20-22]. The present results indicate that the anti-tumour properties of PSK observed in clinical trials might be due to a dual biological activity: 1) a direct cytotoxic activity on tumour cells and 2) an immunomodulatory activity largely produced by NK cell activation. A similar dual activity has also been described in a *Calendula *extract, LACE, which produces an *in vitro *cytotoxic activity and *in *vivo immunomodulatory effect on tumour cell lines, including human and mouse melanioma cells, increasing the number and activation of CD4+, CD19+ and NKT cells [29]. PSK suppressed *in vivo *metastases in spontaneous metastasis assays of mouse fibrosarcoma, melanoma, rat hepatoma AH60C and mouse colon cancer 26 [17,30,31]*via *NK cell activation. Based on the present findings, it can be hypothesised that this anti-metastatic capacity may also derive from the cytotoxic component of PSK.

Research into the biological mechanisms underlying the anti-tumour effect of PSK is ongoing. We can now add a direct cytotoxic effect on tumour cells to the previously described immunomodulatory effect of this polysaccharide. Greater knowledge of the molecular mechanisms implicated in PSK anti-tumour activity may improve cancer immunotherapy, leading to the application of new anti-tumour protocols.

## Conclusion

PSK shows *in vitro *growth inhibition of various tumour cell lines, producing cell cycle arrest/slowing, apoptosis and induction of caspase-3 expression. In combination with IL-2, PSK induces proliferation of PBLs. The biological activity of PSK appears to include both an immunomodulatory effect on NK cells and a cytotoxic effect on tumour cells

## Abbreviations

PBLs: Peripheral Blood Lymphocytes; LACE: laser-activated calendula extract; 7-AAD: 7-amino-actinomicin D; BrdU: 5-Bromo-2-deoxyuridine; Concan.: Concanavalin A; LAK: lymphocyte-activated killer; NK: Natural killer; TMB: tetramethyl-benzidine.

## Competing interests

Materials for these studies were partially supported by a grant from Kureha Chemical Industry (Japan), which manufactures PSK. The authors declare that they have no other competing interest.

## Authors' contributions

EJM, EB and IR performed the assays. IA and AC helped in some experiments. FG and AGL designed the study and drafted the manuscript. All authors have read and approved the final manuscript.

## Pre-publication history

The pre-publication history for this paper can be accessed here:


